# Fibrotic Scar in CNS Injuries: From the Cellular Origins of Fibroblasts to the Molecular Processes of Fibrotic Scar Formation

**DOI:** 10.3390/cells11152371

**Published:** 2022-08-02

**Authors:** Maryam Ayazi, Sandra Zivkovic, Grace Hammel, Branko Stefanovic, Yi Ren

**Affiliations:** Department of Biomedical Sciences, Florida State University College of Medicine, Tallahassee, FL 32304, USA; ma18k@fsu.edu (M.A.); sz18d@fsu.edu (S.Z.); geh17@fsu.edu (G.H.); branko.stefanovic@med.fsu.edu (B.S.)

**Keywords:** fibrotic scar, fibroblasts, spinal cord injury (SCI), traumatic brain injury (TBI), myelin debris

## Abstract

Central nervous system (CNS) trauma activates a persistent repair response that leads to fibrotic scar formation within the lesion. This scarring is similar to other organ fibrosis in many ways; however, the unique features of the CNS differentiate it from other organs. In this review, we discuss fibrotic scar formation in CNS trauma, including the cellular origins of fibroblasts, the mechanism of fibrotic scar formation following an injury, as well as the implication of the fibrotic scar in CNS tissue remodeling and regeneration. While discussing the shared features of CNS fibrotic scar and fibrosis outside the CNS, we highlight their differences and discuss therapeutic targets that may enhance regeneration in the CNS.

## 1. Introduction

Tissue injury activates a cascade of wound healing mechanisms to clear cellular debris, limit further damage, and initiate repair of the injured tissue. In this process, fibroblasts deposit extracellular matrix (ECM) proteins, including fibrillar collagen and fibronectin, which are essential for wound closure and healing. However, excessive and prolonged deposition of these proteins due to an uncontrolled wound healing response leads to fibrosis and fibrotic scar formation that affects the functional restoration of the tissue [1,2]. Apart from fibrosis in injured tissue, fibrosis is common in many pathological conditions, occurring in organs such as the liver [3,4,5,6,7], heart [8,9,10,11,12], kidneys [13,14,15,16,17], lungs [18,19,20,21], and systemic sclerosis [22,23,24,25]. 

Insult to the central nervous system (CNS) that is induced by trauma generates cellular debris, activates resident cells, infiltrates circulating immune cells, and eventually forms two distinct scars: glial scar and fibrotic scar [26]. The glial scar, unique in CNS injuries, is mainly formed by reactive astrocytes, which are characterized by the increased expression of glial fibrillary acidic protein (GFAP), hypertrophy, and the extension of processes [27,28]. These cells surround the lesion and separate the injured area from uninjured tissue. Oligodendrocyte precursor cells (OPCs) [29,30,31,32] and microglia [33,34,35] are also located in the glial scar region. On the contrary, the fibrotic scar is located in the lesion core and is characterized by the presence of fibroblasts and fibroblast-like cells depositing ECM proteins [36]. Even though the CNS glial and fibrotic scars are in close proximity and there is active cross-talk between them [28,37], they are well demarcated with the glial scar on the lesion border and fibrotic scar located inside the injured area. Compared to other organs, the literature on fibrosis in CNS is minimal. The outcomes of fibrosis in the CNS and outside the CNS are similar. Both result in the overproduction of ECM proteins, causing hardening/scarring of tissue and organ failure; however, their path to accomplish this task are quite different. These differences may be assigned to the unique CNS environment [38,39], including the immune-privileged and highly regulated neurovascular unit that is commonly referred to as the blood-brain barrier (BBB) and the blood spinal cord barrier (BSCB) [40,41,42,43]. As a result, the cellular origins and cellular mediators of fibrosis in the CNS are unique; thus, little is known about their implications in CNS fibrosis. This review focuses on the current knowledge surrounding the pathogenesis of fibrosis following trauma to the CNS. We will discuss the cellular origins of fibroblasts, their spatial and temporal distribution, the mechanism of fibrotic scar formation, and the pathological roles of the fibrotic scar. These are all factors that are important in gaining a better understanding of fibrotic scar in CNS and more successful interventional treatments for CNS trauma.

## 2. Fibrotic Scar Components

The components of fibrotic scar in CNS injuries are summarized in Table 1. 

## 3. Timing of Fibrotic Scar Formation in the CNS

Fibrotic scar formation starts in the sub-acute phase of spinal cord injury (SCI), and its maturation continues in the chronic phase [58]. In mouse SCI, fibroblasts start accumulating in the lesion core around 5 days post-injury (dpi), and their number peaks around 7 dpi [36,44,45]. The increased fibroblasts in the lesion correlate with macrophage infiltration to the injury area [45]. As the number of fibroblasts increases, ECM protein deposition becomes prominent in the lesion [36,59]. By 14 dpi, the matured fibrotic scar is formed in the lesion core of the injured spinal cord and brain. This scar remains in the lesion core chronically after injury (56 dpi) [36,45,59,60]. A glial scar surrounds the mature fibrotic scar and infiltrated macrophages. Similar to the fibrotic scar, reactive astrocytes and microglia contribute to the maturation of the glial scar by 14 days after SCI [28,33,61]. 

## 4. The Cellular Origins of Fibroblasts in the Fibrotic Scar

**Figure 1:**
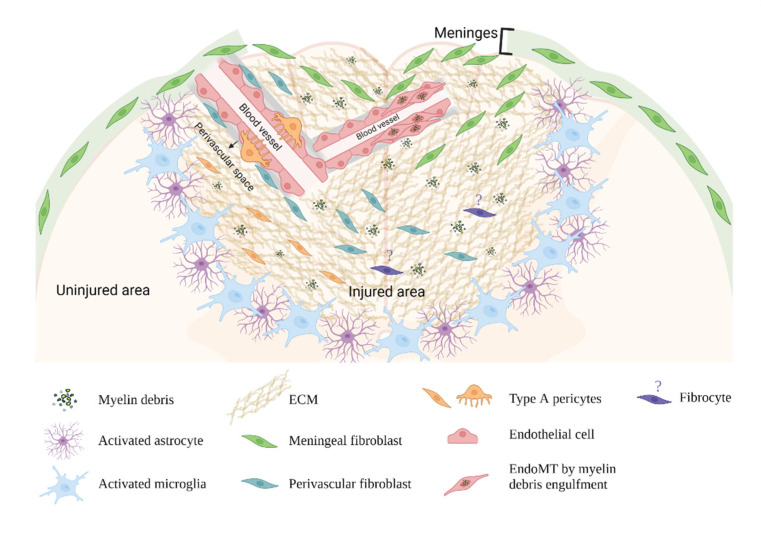
**Origins of fibroblasts in the CNS fibrotic scar.** This figure summarizes the origins of fibroblasts that contribute to the deposition of ECM proteins and the formation of the fibrotic scar in the CNS. This image was created with BioRender.com (accessed on 29 July 2022).

Fibroblasts are cells of the connective tissue that produce ECM proteins and maintain tissue homeostasis. Fibroblasts are activated in injured tissues and differentiate into myofibroblasts, producing growth factors, depositing ECM proteins, and contracting the wound [62,63]. In the CNS, fibroblasts are present in the meninges and choroid plexus at birth. During the first few weeks after birth, they also appear in the perivascular space in the parenchyma [39,64]. In adults’ healthy CNS, the localization of fibroblasts remains limited to meninges, the perivascular space, and choroid plexus [39,65]. However, in CNS injuries, the injury epicenter is filled with fibroblasts, raising the question of where these fibroblasts come from. The origins of fibroblasts in the CNS fibrotic scar are discussed in this review and summarized in Figure 1.

### 4.1. Meningeal Fibroblasts

Meningeal fibroblasts have long been shown to play a role in the fibrotic scar formation in CNS trauma after migrating into the lesion through the torn meninges [37,60,66,67]. Meningeal cells that are co-cultured with astrocytes do not mix and form a structure that is similar to the CNS lesions in vivo with fibroblasts in the core depositing the ECM proteins and astrocytes surrounding them as in the glial scar [68]. Initial reports suggest that if dura, the outermost layer of meninges, remains intact, meningeal fibroblasts cannot migrate into the lesion, and fibrotic scar formation is attenuated [69]. However, a fibrotic scar still forms in the contusive SCI mouse model, in which the dura remains intact. This fibrotic scar has a different morphology compared to the one that is formed in the models in which the dura is damaged [36]. Also, a fibrotic scar is observed in non-traumatic pathologies such as multiple sclerosis (MS) [70], suggesting that fibroblast-like cells from different origins are present in addition to meningeal fibroblasts.

### 4.2. Perivascular Fibroblasts

Perivascular fibroblasts are located in the perivascular space and are loosely attached to the larger blood vessels in the CNS but are absent from the capillaries [65,71]. Perivascular fibroblast contribution to CNS fibrotic scar formation was first described by Soderblom et al. using the Col1α1-GFP mouse model [36]. This transgenic mouse expressing GFP under collagen α1(I) (Col1α1) promotor is a valuable mouse model to study CNS fibroblasts in the mouse models of traumatic brain injury (TBI) and SCI [36,70,71,72]. Following contusive SCI, Col1α1-GFP cells accumulate in the injury epicenter and form the fibrotic scar 2 weeks after SCI. All of these Col1α1-GFP fibroblasts were also positive for platelet-derived growth factor receptor β (PDGFRβ). This study proposed that scar forming fibroblasts originate from perivascular fibroblasts and not meningeal fibroblasts because the dura remained intact in contusive SCI and Col1α1-GFP cells were observed leaving the blood vessels and migrating toward the fibrotic scar [36]. Moreover, in a rat model of brain injury that was induced by 3-nitropropionic acid (3-NP), cells expressing PDGFRβ, located abluminal to cells expressing smooth muscle actin (αSMA) on larger blood vessels, resembling perivascular fibroblasts spread to the extravascular area within the lesion core 14–28 days post-3-NP injection. Processes of PDGFRβ-expressing cells form a mesh that colocalizes with collagen fibrils [73]. In experimental autoimmune encephalomyelitis (EAE) lesions, CNS fibroblasts expressing Col1α1 are the main cells that are responsible for fibrotic scar formation. However, the partial contribution of perivascular fibroblasts versus meningeal fibroblasts cannot be distinguished as both express Col1α1 [47,70]. 

Despite these observations regarding the fibrotic scar formation by perivascular fibroblasts, the molecular identity and morphology of perivascular fibroblasts have only been recently characterized by single-cell RNA-sequencing (scRNA-seq) and in vivo two-photon microscopy [65,71,74,75]. They are cells with flattened somata and thin ruffled processes. Perivascular fibroblasts are enriched for transcripts that encode ECM proteins such as fibrillar and non-fibrillar collagens, ECM modifiers, and ECM receptors [65,71]. Moreover, scRNA-seq identified two subtypes of perivascular fibroblasts (Type I and II) in mice and three subtypes in the human brain (Type I, II, and III) [65,75]. Based on the transcriptional analysis in healthy humans, Type I perivascular fibroblasts are likely the primary subtype that drives the fibrotic scar formation after CNS injuries [75]. More investigation is required to determine the contribution of perivascular subtypes in fibrotic scarring.

### 4.3. Pericytes

Pericytes are perivascular cells covering blood vessels on the capillaries and venous vasculature [74,76]. In contrast to perivascular fibroblasts, pericytes are firmly attached to blood vessels as they are embedded in the basement membrane of the vessels [65]. They are identified by various markers such as platelet-derived growth factor receptor α (PDGFRα), PDGFRβ, αSMA, desmin, CD13, and neuron-glial antigen 2 (NG2). Although these markers are not pericyte-specific and overlap with other perivascular cells, such as vascular smooth muscle cells and perivascular fibroblasts, they have been widely used in pericyte studies as pericyte markers [77,78]. Observations regarding pericyte-derived fibrotic scarring have been mainly based on the above-mentioned marker expression. Pericyte-derived fibrotic scar has been reported in brain injury [79] epileptogenesis [80], experimental ischemic lesions, and human stroke lesions [81], as well as in other tissues and organs, including dermal fibrosis [82], kidney fibrosis [83,84,85], skeletal muscle fibrosis [86], and liver fibrosis [87].

A subpopulation of pericytes, known as Type A pericytes, has been suggested as fibroblasts’ origin in pathological scarring after SCI, TBI, and EAE [44,46]. Type A pericytes proliferate and leave the blood vessels, deposit ECM proteins, and contribute to fibrotic scar formation in CNS injuries. While Type A pericytes are identified based on the expression of Glast, an astrocyte marker, they also express PDGFRα, PDGFRβ, and CD13 [44,46,88]. Type A pericytes are abluminal to Type B pericytes, with Type B expressing desmin and αSMA in addition to other pericyte markers [44]. Despite these observations and many other studies regarding different subtypes of pericytes [89], a recent scRNA-seq study did not identify pericytes subtypes in mouse brain vasculature [65]. However, two distinct subtypes have been identified in the human brain by scRNA-seq [74]. Further investigations are required to determine the role of pericytes subtypes in CNS fibrotic scarring. 

### 4.4. Endothelial Cells

Endothelial cells are the innermost layer of cells forming the blood vessels. Endothelial cells can transition to fibroblast-like cells through the process of endothelial to mesenchymal transition (EndoMT) [90,91]. EndoMT is a process in which endothelial cells lose their characteristic endothelial features and acquire mesenchymal properties [92,93]. During EndoMT, the expression of endothelial markers such as vascular endothelial cadherin (VE-cadherin), CD31, von Willebrand factor (vWF), and platelet endothelial cell adhesion molecule-1 (PECAM-1) are reduced, and the expression of mesenchymal markers such as αSMA and vimentin are increased [91,94,95,96]. In addition, endothelial cells transition towards a mesenchymal phenotype by acquiring spindle shape morphology and contractibility [97,98], becoming migratory, and demonstrating increased ECM protein expression [99,100]. Transforming growth factor (TGF)-β acts as a master regulator of EndoMT [101] and is the primary cytokine driving the formation of fibrotic scars in the injury site [102,103]. 

Despite existing studies of EndoMT and endothelial-derived fibroblasts in different tissues [104,105,106,107], it is unclear whether they contribute to CNS fibrotic scar formation. A study in our lab has proposed endothelial cells as a possible cellular origin of fibroblast-like cells in fibrotic scar after SCI [108]. This study showed that engulfment of myelin debris induces changes in endothelial cell characteristics that resemble EndoMT. Moreover, engulfment of myelin debris increased TGF-β1 expression, and specific blockage of TGF signaling abrogated myelin debris-induced EndoMT. Autophagic processing of engulfed myelin debris is crucial for myelin debris-induced EndoMT because the depletion of *Atg5* in endothelial cells failed to induce EndoMT. In vivo, EndoMT is also observed in the lesion core after SCI and in EAE. This study demonstrated that endothelial cells that undergo EndoMT following myelin debris engulfment and processing could differentiate into fibroblast-like cells that contribute to fibrotic scar formation in SCI lesions [108]. 

Moreover, a study investigating BBB dysfunction in experimental animal models of stroke, MS, seizure, and TBI also suggested a potential role of endothelial cells in fibrotic scar formation [109]. Based on these studies, transcription of some ECM proteins and ECM modulators such as collagens, extracellular proteases, and extracellular protease inhibitors are upregulated in brain endothelial cells with EndoMT implicated in these disease conditions [109]. EndoMT is observed in human post-mortem MS brain tissues [110] and stroke lesions in a mouse model [111]. Therefore, endothelial cells may be a common cellular origin of fibroblast-like cells in CNS demyelinating diseases. More investigations are required to understand the contribution of endothelial cells to CNS fibrosis.

### 4.5. Circulating Blood Fibrocytes

Circulating blood fibrocytes are bone marrow-derived mesenchymal progenitors that simultaneously express stem cell markers, such as CD34, monocyte markers, such as CD45, and have some fibroblasts characteristics, such as an active collagen I and fibronectin synthesis [112]. Circulating blood fibrocytes migrate to the injured tissue following injury and/or inflammation and differentiate into fibroblasts [113,114]. Fibrocytes contribute to fibrotic reaction in skin wounds [115], atherosclerosis [116], liver fibrosis [117], cardiomyopathy [118], and lung fibrosis [119]. Fibrocytes in the CNS have been reported in fibrotic wall formation in brain abscesses. During a pathogenic infection in the brain, collagen and fibronectin deposition form a fibrotic wall to encapsulate the abscess and prevent its spread to healthy parenchyma [120]. In the EAE model, fibrotic scarring is mainly attributed to the proliferating CNS fibrocytes and not circulating blood fibrocytes [47]. It remains unclear whether fibrocytes are involved in forming CNS fibrotic scars following sterile injuries such as SCI and TBI.

### 4.6. Approaches for Studying the Origins of Fibroblasts in the Fibrotic Scar

Based on the studies that are discussed above, single approaches are insufficient to accurately determine the origin of fibroblasts in the CNS fibrotic scar. This is primarily because perivascular fibroblasts and mural cells have a similar marker expression and are located near each other [39]. Therefore, immunostaining or using a single transgenic mouse line cannot determine the origin of fibroblasts within the fibrotic scar. A combination of approaches as used by Dorrier et al. to determine fibroblasts’ origin in EAE mouse model must be used to avoid confusion in interpreting experimental results [47]. These approaches include: first, lineage tracing using different transgenic mouse lines to exclude mural cells as fibroblasts’ origin; second, using bone marrow chimeric mice to exclude fibrocytes as fibroblasts’ origin; and finally, scRNA-seq to determine the molecular identity of fibroblasts within the fibrotic scar more precisely [47]. 

## 5. The Mechanism of Fibrotic Scar Formation

Organ fibrosis results from a persistent tissue healing response which starts with an inflammatory response and macrophage infiltration into the damaged tissue to clear cellular debris after trauma to the organ. Immune cells are polarized toward the healing phenotype (M2 macrophages and Th2 lymphocytes) and secrete profibrotic factors activating fibroblasts to deposit ECM proteins, such as fibrillar collagens. If this healing response persists, the continuous activation of fibroblasts and deposition of ECM proteins creates stiff scar tissue, which is different from the original tissue structure. Alternatively, during normal wound healing, fibroblasts undergo apoptosis, collagen is turned over during ECM remodeling, and pathological scarring is absent [2,121]. 

Similar to fibrosis outside the CNS, the inflammatory response and macrophage infiltration are involved in fibroblast accumulation in CNS injuries. In the mouse SCI model, both fibroblasts and infiltrating macrophages reside in the lesion core, and there is a timely correlation between the presence of fibroblasts and infiltrating macrophages [45]. The reduction of circulating macrophages in the injured spinal cord reduced the density of fibroblasts at 7 and 14 days after SCI, providing evidence that infiltrating macrophages are involved in recruiting fibroblasts to the lesion core [45]. In the EAE mouse model, the infiltration of T lymphocytes and macrophages is followed by fibroblast accumulation, implicating that the inflammatory response orchestrates fibrotic scar formation in MS [47]. 

The mechanism of fibroblast activation to secrete ECM proteins is not well understood in the CNS. However, similar mechanisms and principles in other organ fibrosis may activate fibroblasts and initiate fibrotic scar formation in the CNS. In general, fibroblasts can be activated in three ways: (1) profibrotic cytokine-induced activation, (2) direct activation, and (3) self-activation [2]. 

### 5.1. Fibroblast Activation by Profibrotic Cytokines

Profibrotic cytokines such as TGF-β and platelet-derived growth factors (PDGFs) are mainly secreted by M2 macrophages [122,123,124,125,126], and their major roles are to recruit and activate fibroblasts [127,128]. 

#### 5.1.1. TGF-β 

TGF-β is the most potent profibrotic cytokine in fibroblast activation in different tissues [129,130]. For a review of the TGF-β signaling pathway and its role in fibrosis, we refer to [131,132]. Elevated levels of TGF-β1 and TGF-β2 are seen in CNS injuries [133,134,135,136,137]. Local injection of recombinant TGF-β into the injured brain in rats increases the deposition of fibronectin and laminins [102]. On the contrary, the local injection of anti-TGF-β1 [102] or anti-TGF-β2 antibodies [138] into the injured brain attenuates fibrotic scar formation, as depicted by the absence of laminins and fibronectin. While these studies show the importance of TGF-β in CNS fibrotic scar formation, it is also essential to identify TGF-β-producing cells and their target cells. Microglia/macrophages are the major source of TGF-β1 in ischemia, MS, TBI, and SCI [134,137,139,140]. TGF-β1 is also produced by astrocytes [137,141], meningeal fibroblasts [140], and neurons [137]. The identification of cells that are expressing TGF-β receptors in CNS lesions can reveal the target cells of this pathway. TGF-β receptors are not abundantly expressed in the normal brain [60]. However, in active demyelinating regions of MS, both TGF-β Types I and II receptors (TGF-βR I and II) are expressed on macrophages, hypertrophic astrocytes, and endothelial cells [141]. In TBI lesions, TGFβ-RI and TGFβ-RII are expressed in meningeal fibroblasts [60]. Therefore, TGF-β can bind to TGFβ receptors on the surface of macrophages, astrocytes, endothelial cells, and fibroblasts during CNS injuries to initiate transmembrane signaling that promotes fibrosis. Moreover, TGF-β has been identified as one of the main pathways promoting EndoMT, resulting in more accumulation of ECM-depositing fibroblasts and increased fibrosis [104,142,143,144,145,146]. As mentioned earlier in the review, myelin debris, which is abundantly present in CNS injuries [147,148] and other demyelinating diseases [149,150], increases TGF-β expression in endothelial cells upon its engulfment. This TGF-β expression is followed by EndoMT, likely through autocrine signaling [108]. Future studies will continue to provide more understanding of the role of TGF-β in CNS fibrosis. 

#### 5.1.2. PDGFs 

PDGFs are dimers of A and B polypeptides with different isoforms, namely PDGF-AA, PDGF-BB, PDGF-CC, PDGF-DD, and PDGF-AB [151]. PDGFs, particularly PDGF-BBs, are the most critical profibrotic factors that stimulate fibroblast proliferation and migration [129,152,153,154,155,156]. In liver fibrosis, PDGF-BB is a prominent profibrotic cytokine that is secreted by resident cells and infiltrating immune cells [157,158]. The secreted PDGF-BB stimulates the proliferation and activation of hepatic satellite cells (HSCs) into myofibroblasts causing these cells to be major depositors of ECM proteins during fibrosis [159]. PDGF receptors (PDGFRs) are Class III receptor tyrosine kinases that have three dimeric forms (-αα, -ββ, and -αβ) [156,160]. PDGF-BB has a high affinity for PDGFRαα, PDGFRαβ, and PDGFRββ, while PDGF-AA has a high affinity for dimeric PDGFRαα [161]. The high affinity of PDGF-BB to all three PDGFRs makes this cytokine a more potent chemoattractant for myofibroblasts than PDGF-AA [162]. PDGF-BB/PDGFRβ signaling is involved in liver [163], myocardial [164], and lung fibrosis [165,166]. PDGF-DD, similar to PDGF-BB, has an affinity for PDGFRβ [167]. PDGF-AA is involved in accelerating cell recycling and inducing fibroblast proliferation [168]. In cutaneous wound healing, PDGF-AA that is secreted by senescent fibroblasts and endothelial cells stimulates myofibroblast differentiation and wound closure [169]. Neutralizing PDGFRs with antibodies showed decreased PDGF-AA and -BB-induced collagen I deposition in dermal and cardiac fibrotic tissue [170,171,172]. It also decreased the migration of PDGF-stimulated cells in dermal and lung fibrosis [170]. Prominent fibrotic scar tissue with PDGFRβ^+^ cells is observed in CNS injuries, implicating the importance of the PDGF signaling pathway in CNS fibrosis [46,173]. A recent study showed that the expression of PDGF-BB and PDGF-DD is increased in the mouse SCI model. The exogenous injection of PDGF-BB and PDGF-DD induces fibrosis in the normal spinal cord, which can be attenuated by a PDGFRβ inhibitor. Therefore, PDGF-BB and PDGF-DD can induce fibrotic scarring by activating PDGFRβ. Astrocytes are identified as the main producer of PDGF-BB, while macrophages/microglia and fibroblasts are the main source of PDGF-DD in SCI lesions [173].

In addition to TGF-β and PDGFs, some cytokines can enhance fibrotic scar formation indirectly by recruiting macrophages. For example, tumor necrosis factor (TNF) ligand superfamily member 13 (TNFSF13), also known as APRIL, is involved in macrophage recruitment to SCI lesion. In APRIL KO mice, fibrotic scarring following SCI is reduced [174]. Therefore, APRIL promotes fibrotic scar formation indirectly by promoting acute inflammatory response and recruiting macrophages to the lesion core [174]. 

### 5.2. Direct Activation of Fibroblasts

Necrotic cells that are generated from tissue damage release damage-associated molecular patterns (DAMPs) such as histones, heat shock proteins, DNA, and RNA [175]. In vitro treatment of fibroblasts with necrotic myocardial cells can increase fibroblast proliferation and migration by activating Toll-like receptor 4 (TLR4) [176]. The injection of necrotic myocardial cells into the mouse heart induces myocardial fibrosis in a TLR4-dependent manner [176]. TLR4 activation in fibroblasts increases sensitivity to TGF-β and collagen I expression in vitro [177]. The direct activation of fibroblast by DAMPs in CNS injuries is yet to be investigated. 

### 5.3. Mechanical Activation (Self-Activation)

In organ fibrosis, collagen cross-linking increases tissue stiffness and renders fibrosis irreversible [178,179]. In cardiac fibrosis, tissue stiffness further activates fibroblasts [180,181]. Fibroblast activation by tissue stiffness can create a self-activation loop that triggers fibrosis [2]. Atomic force microscopy and elastic modulus measurements have shown that the lesion site in rat models of TBI and SCI is less stiff than controls, with some increase in stiffness in the injured lesion tissue occurring 3 weeks after injury yet remaining softer than the control’s tissue [182]. However, the lesion site is significantly stiffer at 12 weeks after mouse contusion SCI, suggesting that tissue stiffening in the injured CNS is present in the chronic lesions but not in the acute or sub-acute phases of injury [183]. Therefore, fibroblasts in CNS injuries may be activated by the stiffness of the tissue in the chronic phase. 

## 6. Potential Role of Fibroblasts in Neuroinflammation 

Fibroblasts and cells that can transition into fibroblast-like cells, such as pericytes and endothelial cells, have multiple properties to mediate the inflammatory response. First, they can activate the innate and adaptive immune systems upon sensing DAMPs. For example, NG2^+^ pericytes that are activated by DAMPs and pathogen-associated molecular pattern molecules (PAMPs) increase the expression of C-X-C motif chemokine ligand 1 (CXCL1), CXCL8, macrophage migration inhibitory factor (MIF), CCL2, and interleukin 6 (IL-6) that attract the infiltration of immune cells such as monocytes and neutrophils. MIF that is released from activated pericytes is sufficient to increase the expression of TLRs, integrins, and matrix metalloproteinases (MMPs) in immune cells [184]. Similarly, 2 h after a systemic infection that is caused by intraperitoneal injection of LPS, cells resembling perivascular fibroblasts expressing PDGFRβ, Col1α1, and regulator of G protein signaling 5 (RGS5) increase the expression of CCL2 in the brain [183]. In addition to innate immune responses, fibroblasts are also involved in the adaptive immune response. Fibroblastic reticular cells (FRCs) regulate the migration pattern of T-cells in the lymph nodes [185]. Upon exposure to TNF-α and interferon (IFN)-γ, CNS pericytes are activated, and the expression of major histocompatibility complex II (MHC-II) is increased, suggesting that the activated pericytes can act as antigen-presenting cells to activate T lymphocytes [186]. 

Secondly, fibroblast/fibroblast-like cells can enhance immune cell accumulation by increasing cell adhesion. For example, a subset of cancer-associated fibroblasts (CAFs) produce fibroblast activation protein-α (FAP) which increases macrophage adhesion via scavenger receptor A [187]. In another example, the activation of NG2^+^ pericytes by DAMPs and PAMPs results in a significant increase in the expression of intercellular adhesion molecule 1 (ICAM1), which enhances the adhesion of macrophages and neutrophils [184]. Vascular cell adhesion molecule 1 (VCAM-1) and ICAM-1 that is produced by brain pericytes also facilitate pericytes-T-cell interactions [186,188]. 

**Figure 2:**
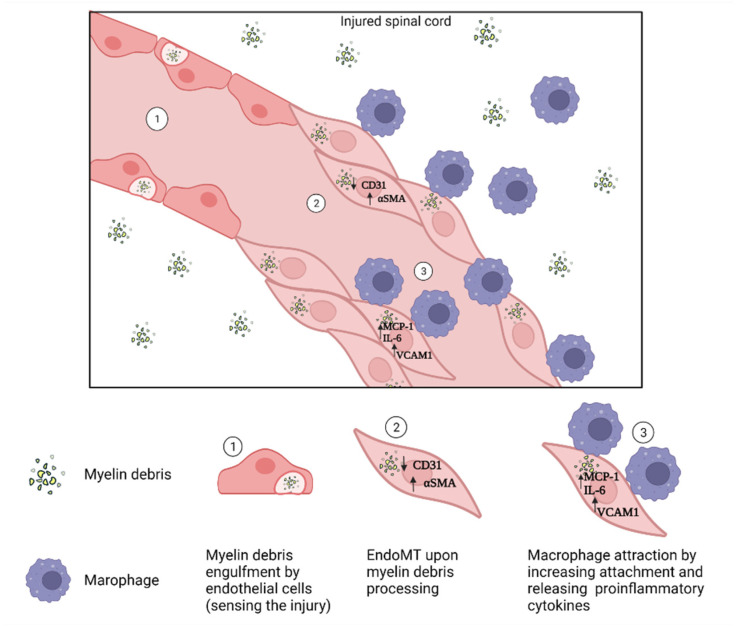
**Role of endothelial cells in neuroinflammation in SCI lesions.** Upon SCI, ongoing demyelination of axons causes the persistent presence of myelin debris in the injury area. Endothelial cells sense the injury and engulf the myelin debris, resulting in EndoMT. These endothelial cells secrete inflammatory factors that induce macrophage infiltration and attachment, promoting neuroinflammation. This image was created with BioRender.com (accessed on 29 July 2022).

Therefore, some fibroblasts/fibroblast-like cells can elicit the immune responses by sensing the injury, releasing cytokines to recruit innate immune cells, activating the adaptive immune system, and increasing adhesion for immune cell infiltration and activation. According to the characteristics that are discussed above, fibroblasts/fibroblast-like cells that are derived from meningeal cells, endothelial cells, pericytes, and perivascular cells can potentially play a role in neuroinflammation in CNS injuries. An example is the engulfment of myelin debris by endothelial cells (sensing the injury), where endothelial cells transition toward a fibroblast-like phenotype and overexpress important inflammatory molecules such as CCL2, IL-6, and VCAM-1 (immune response) [108] (Figure 2). 

## 7. Fibrotic Scar in CNS Tissue Remodeling and Regeneration

Remodeling is the critical phase of tissue repair in which unwanted cells in the granulation tissue undergo apoptosis, the ECM is reorganized, and tissue-specific cells grow into the lesion to replace the lost cells and start the regeneration [189]. However, chronic inflammation and persistence of fibroblasts for an extended period will lead to excessive ECM protein deposition and scar formation, negatively affecting tissue remodeling and regeneration [189]. In general, the fibrotic scar is the cause of pathologic tissue remodeling and is an impediment to axonal regeneration in the chronic phases of different CNS diseases and injuries such as stroke, MS, SCI, and TBI. The fibrotic scar does this through the production of inhibitory molecules and establishing a physical barrier that prevents the regeneration of axons through the lesion area [26,190,191,192]. 

One early study showed that axons grow into a glial scar within a brain lesion, but the growth is then terminated once they reach the fibrotic scar [193]. The inhibition of fibrotic scarring in the injured brain of rats by collagen neutralizing antibodies or inhibiting collagen synthesis by administering an iron chelator allows axon regeneration, indicating that the fibrotic scar is an obstacle to regeneration [194,195]. Similarly, delaying or reducing fibrotic scar formation in SCI by an iron chelator [196], decorin treatment [197], or microtubule stabilization [198], enhanced axonal growth and regeneration. After SCI, inhibiting the proliferation of Type A pericytes reduces scar formation and ECM protein deposition, promoting axonal regeneration and improving functional recovery [192]. Reducing fibrotic scarring in the EAE mouse model enables more oligodendrocyte lineage cells to migrate into the lesion core and reduces the worsening of motor abilities [47]. Although reducing the fibrotic scar is beneficial in axonal regeneration and functional recovery, it is not enough to impede demyelination and promote remyelination [47]. Other than being a physical barrier, the fibrotic scar contains various molecules that can inhibit axonal growth including phosphacan, NG2, tenascin-C [56], and semaphorin III [57,199]. Various studies have shown that eliminating these different inhibitory molecules enhances axonal regeneration and, in some cases, promotes functional recovery after TBI and SCI [200,201,202]. 

Even though the fibrotic scar’s dense matrix acts as a physical barrier, preventing axonal growth, ECM proteins do not inherently have an inhibitory growth effect on axons. For example, between 7–14 dpi in rat SCI, axonal sprouting into the lesion core was associated with collagen IV, suggesting that collagen IV is not an inhibitory factor for axon regeneration [203,204]. The association of regenerating axons with laminins in SCI also shows its non-inhibitory effect on axonal growth [204]. Biomaterial-based scaffolds such as collagen scaffolds have been used in CNS injury treatment to support cell attachment and guided growth. In addition, factors for enhancing axonal regeneration, including vectors for gene therapy, can be incorporated into these scaffolds [205,206]. In rat SCI, a polylaminin-based scaffold enhances regeneration and motor function recovery [207]. A fibronectin mat that is placed on the injury site, or a dissolved fibronectin mat that is injected into the injured spinal cord of rats, decreased apoptosis at the lesion site, suggesting a neuroprotective role of fibronectin [208]. The systemic administration of synthetic fibronectin peptides has also demonstrated a neuroprotective role in neural transplantation in the rat brain [209]. These studies suggest that collagen I and IV, fibronectin, and laminins do not inhibit axon regeneration; however, their influence on the proliferation and migration of other types of cells is of great interest. For example, collagen I significantly reduce the migration of OPCs in vitro, while fibronectin and laminins increase their migration [47].

Interestingly, in studies that inhibited the formation of the fibrotic scar in SCI, there was a complete failure in the sealing of the injury site and it had an overall negative impact on tissue integrity [44]. In neonatal mouse SCI, collagen I and laminin deposition are almost absent, and fibrotic scar fails to form. Under these conditions, microglia grow into the lesion, depositing fibronectin into the lesion core and creating a bridge between the two wound edges. This transient fibronectin expression by microglia at 3 dpi is crucial for the nearly complete recovery that was observed in neonatal mice [210]. This study highlights that the fibrotic scar wound healing mechanism is essential for closing the injury site and maintaining tissue integrity. Reducing, rather than eliminating the fibrotic scar, or reducing a specific isoform of fibronectin that is found in chronic fibrotic scarring after SCI in mice, enhances axon growth and functional recovery [44,192]. This demonstrates that while fibrotic scar formation can lead to pathologic tissue remodeling and the prevention of axonal regeneration in many different CNS diseases and injuries, it also plays a critical role in wound closure. The dynamic nature of fibrosis should be further investigated in CNS pathology. 

## 8. Therapeutic Targets for Fibrotic Scar 

### 8.1. Targeting Macrophages

Macrophages play an essential role in fibroblast activation and fibrotic scar formation. Intraperitoneal injection of clodronate liposome reduces macrophage recruitment following SCI, thus, reducing fibrotic scar formation and enhancing axonal growth [45]. Intravenous injection of immune-modifying nanoparticles such as carboxylated poly lactide-co-glycolide (PLGA) reduced macrophage recruitment to the lesion site, thus, reducing fibrotic scarring, increasing axonal density, and enhancing functional recovery in SCI mice [211]. In mice lacking the ECM protein periostin, decreased macrophage infiltration and reduced proliferation of PDGFR-β-expressing pericytes in the lesion site are associated with reduced fibrotic scar formation, resulting in improved functional recovery after SCI [212]. These studies indicate the importance of inflammation regulation on fibrosis and functional recovery. 

### 8.2. Targeting Fibroblasts and Fibroblast-like Cells

The fibrotic scar that is formed by meningeal fibroblasts can be reduced by repairing the damaged dura. This approach, referred to as duraplasty, is performed by planting a dura tissue graft at the lesion site. In rat SCI, duraplasty reduces fibrotic scarring and is characterized by reduced basement membrane deposition (monitored by collagen IV and laminin immunostaining) in the scar tissue [69,213]. Dura repair reduces inflammation, inhibits cystic cavitation, and enhances the functional recovery within rat SCI lesions [213,214]. Duraplasty not only reduces scarring but also reduces the pressure on the swollen damaged spinal cord [215]. Although duraplasty showed beneficial results, it is insufficient to significantly improve the functional outcomes in a pig SCI model [216] and human patients [217]. 

As mentioned earlier in this review, Glast-expressing pericytes (Type A) have been demonstrated to proliferate and contribute to CNS fibrotic scar formation [44,46]. Therefore, these cells are a possible target to reduce fibroblast proliferation in the fibrotic scar. The deletion of the *Ras* gene, specifically in Type A pericytes, inhibits their proliferation and reduces ECM protein deposition and fibrotic scar formation after SCI. This study suggests that targeting pericyte-induced fibrosis may be a promising therapeutic option to improve axon regeneration and functional recovery after SCI [192].

### 8.3. Targeting Profibrotic Cytokines

TGF-β is the master regulator of fibrosis and, therefore, is a target for excessive scarring in CNS fibrosis. The local injection of TGF-β neutralizing antibody through an intraventricular cannula for 10 days after injury attenuates fibrotic scar formation in rat TBI [102,138]. The inhibition of TGF-β signaling in the injured mouse brain by infusing LY-364947, a small molecule inhibitor of TGFβ-RI, attenuates fibrotic scar formation and improves regeneration [103]. The inhibition of TGF-β signaling promotes neuron survival, axon growth, and functional recovery after TBI and SCI [103,218,219,220]. Since macrophages are a significant source of TGF-β in CNS lesions [134,137,139,140], targeting macrophages may control TGF-β expression consecutively. However, Moon and Fawcett showed that reducing scar formation by inhibiting TGF-β1/2 is insufficient to promote axon regeneration in TBI [221]. Although TGF-β is a master regulator of fibrotic scar formation, its inhibition can affect CNS functions. For example, inhibiting TGF-β signaling could have detrimental effects because TGF-β is neuroprotective in CNS lesions. Additionally, TGF-β is a key regulator in the induction of M2 polarization, a macrophage phenotype that is important in tissue repair [222,223,224]. Therefore, by inhibiting TGF-β signaling, one would suppress its role in fibrosis at the expense of its neuroprotective and anti-inflammatory properties. 

PDGFs are profibrotic cytokines and are, therefore, a therapeutic target to control CNS fibrosis. In mouse SCI, the inhibition of PDGFRβ by intrathecal administration of PDGFRβ inhibitor, SU16f, reduces fibrotic scarring, inflammation, and lesion size. This is followed by enhanced axon regeneration and improved functional recovery [173]. 

### 8.4. Targeting Collagen

Targeting constituents of the ECM provides another therapeutic avenue for managing excessive fibrotic scarring. For example, targeting prolyl 4-hydroxylase (PH), a key enzyme in the collagen synthesis process, can aid in disrupting an excessive fibrillar collagen concentration within the scar. An iron chelator can inhibit PH as iron is required for its enzymatic activity [225]. In a rat transection SCI model, the local administration of an iron chelator, BPY-DCA, and cAMP reduced fibrotic scar formation and enhanced axon regeneration and functional recovery [196,226]. The local administration of another clinical iron chelator, deferoxamine mesylate (DFO), reduced fibrotic scarring in a rat SCI model [227].

Fibrillar collagens are the most important ECM constituents of any scar tissue. Fibrillar collagens can self-assemble in vitro but the two major assembly pathways in vivo include direct β1 integrin-mediated assembly and fibronectin-dependent assembly [167,228]. The inhibition of fibril formation by the administration of a monoclonal antibody that binds to the C-terminal telopeptide of the collagen α2(I) leads to degradation of free collagen and controls excessive scarring in a keloid model [229]. 

Targeting heat shock proteins (HSPs) is another therapeutic approach for inhibiting excessive collagen deposition within the fibrotic scar. HSPs are intracellular proteins that are expressed in high concentrations when there is an injury. In SCI, HSPs are involved in modulating secondary injury [230]. The role of HSPs in CNS fibrosis has not been explored, but one HSP that is known as HSP47 is involved in fibrosis outside of CNS. HSP47 is located in the endoplasmic reticulum and is involved in the correct folding of procollagen molecule and, therefore, collagen triple helix formation [231,232]. HSP47 is overexpressed in fibrotic tissues [233], and inhibiting its expression attenuates collagen production and improves fibrosis pathology [234,235]. Interestingly, a Phase 2 clinical trial is currently testing a lipid nanoparticle, ND-L02-s0201, containing siRNA to silence HSP47 in subjects with idiopathic pulmonary fibrosis (Clinicaltrial.gov, accessed on 29 July 2022, NCT03538301). This clinical study raises an exciting possibility of ND-L02-s020’s potential in attenuating collagen production in a CNS injury (i.e., SCI) and improving regeneration. 

## 9. Conclusions

Inflammation, fibroblast activation, and ECM protein deposition are all part of the wound healing process. However, an escalation of this process leads to the formation of a fibrotic scar that hampers regeneration [189]. In this review, we discussed fibrotic scar formation in CNS injuries with information covering pathological fibroblasts’ origins and the mechanism of fibroblast activation. We reviewed how a CNS fibrotic scar is an obstacle for regeneration, and a transient repair response is more favorable for regeneration [210]. We finally discussed the known therapeutic targets for the fibrotic scar. There are still many gaps in our knowledge regarding fibrotic scarring in the CNS, such as details surrounding the mechanisms of fibrotic scarring. Fibroblasts’ interactions with other cells within the lesion are not well explored and also require further investigation. We refer to [236] to review current knowledge on fibroblasts’ cross-talk with other cells after SCI. Furthermore, whether fibroblasts contribute to the neuroinflammation that is present chronically in CNS injuries remains largely unknown [61]. Filling these knowledge gaps will lead to better intervention strategies for the CNS fibrotic scar.

## Figures and Tables

**Table 1:** This table summarizes the critical components that are found in the CNS fibrotic scar.

Fibrotic Scar Components	Specific Integrant	Reference
Fibroblast/Fibroblast-like cells		
	--	[36,44,45,46,47]
	
ECM	Collagen I, IV	[48,49,50,51]
	Fibronectin	[36,52,53,54]
	Laminins	[36,54]
Others	EphB2	[37,55]
	Phosphacan	[56]
	NG2	[56]
	Tenascin	[56]
	Semaphorin III	[57]

## Data Availability

Not applicable.
